# Magnetoreception in birds

**DOI:** 10.1098/rsif.2019.0295

**Published:** 2019-09-04

**Authors:** Roswitha Wiltschko, Wolfgang Wiltschko

**Affiliations:** FB Biowissenschaften, Goethe-Universität Frankfurt, Frankfurt am Main, Germany

**Keywords:** radical pair processes, flavin adenine dinucleotide cycle, superparamagnetic magnetite, trigeminal nerve, magnetic pulse, radiofrequency fields

## Abstract

Birds can use two kinds of information from the geomagnetic field for navigation: the direction of the field lines as a compass and probably magnetic intensity as a component of the navigational ‘map’. The direction of the magnetic field appears to be sensed via radical pair processes in the eyes, with the crucial radical pairs formed by cryptochrome. It is transmitted by the optic nerve to the brain, where parts of the visual system seem to process the respective information. Magnetic intensity appears to be perceived by magnetite-based receptors in the beak region; the information is transmitted by the ophthalmic branch of the trigeminal nerve to the trigeminal ganglion and the trigeminal brainstem nuclei. Yet in spite of considerable progress in recent years, many details are still unclear, among them details of the radical pair processes and their transformation into a nervous signal, the precise location of the magnetite-based receptors and the centres in the brain where magnetic information is combined with other navigational information for the navigational processes.

## 1. Introduction

The magnetic field of the Earth provides animals that can sense it with navigational information: the vector indicates directions, and magnetic intensity and inclination, which decreases from the magnetic poles to the magnetic equator, and possibly also magnetic declination could be used as components of the navigational ‘map’. Birds make use of the geomagnetic field in two ways: the vector provides them with a compass, and other parameters, probably magnetic intensity, appear to be an important component in the navigational ‘map’ for long-distance navigation. How birds sense these parameters is not yet completely understood, although numerous aspects of the receptive processes have been described in recent years.

## 2. Magnetic compass: starting out with radical pair processes

Behavioural experiments, mostly based on the orientation of migratory birds, revealed three surprising characteristics of the avian magnetic compass, indicating a mode of magnetoreception that is basically different from the way that our technical compass shows directions (e.g. [1]).

**Figure 1.**
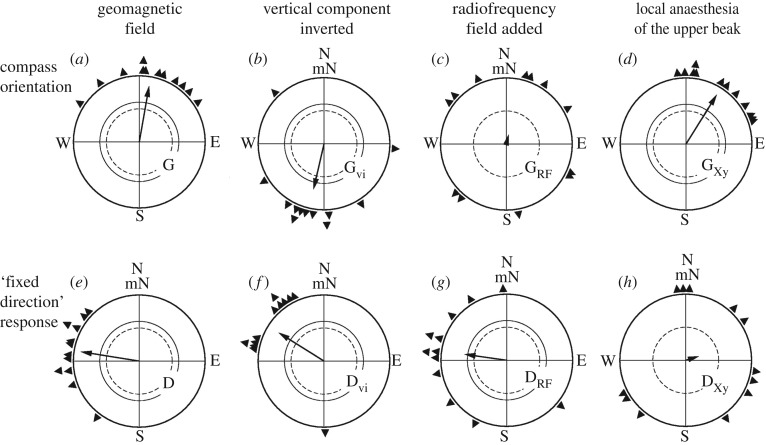
Magnetic compass orientation under green light (G) originating in radical pair processes (*a*–*d*), and a ‘fixed direction’ response in total darkness (D), probably originating in magnetite-based receptors in the beak region (*e*–*h*). The compass response is not sensitive to the polarity of the magnetic field, but to the axial course of the field lines; it is disrupted by radiofrequency fields, but not affected by anaesthesia up the upper beak. The ‘fixed direction’ response, in contrast, is a polar response; it is not affected by radiofrequency fields, but is disrupted by anaesthesia of the upper beak. The triangles at the periphery of the circle mark the mean directions of individual birds, the arrows indicate the grand mean vector and the inner circles give the 5% (dotted) and the 1% significance border of the Rayleigh test (see [2]).

(1) It is an *inclination compass*, not sensitive to the polarity of the magnetic field; instead it senses the axial course of the field lines and interprets their inclination in space (figure 1*a*,*b*; [2–4]). It thus does not distinguish between magnetic North and South, but between ‘poleward’, where the field lines run downward, and ‘equatorward’, where they run upward.
(2) It is narrowly tuned to the intensity of the ambient magnetic field; fields with markedly lower or higher (!) intensity cause disorientation. Yet this ‘functional window’ is not fixed; it can be modified by exposing birds to intensities outside this window, which enables them to use these intensities subsequently for directional orientation [5–7].
(3) It requires short-wavelength light. Tests under near-monochromatic lights revealed that orientation is possible under light from ultraviolet to about 565 nm green; under yellow and red light, birds are disoriented [8–12].

The avian magnetic compass seems to measure directions with an accuracy of about 3° in the vertical [13] and at least 5° in the horizontal [14].

### 2.1. The radical pair model

These properties of the avian magnetic compass caused Ritz and colleagues [15] to propose the radical pair model: upon absorption of a photon, radical pairs are formed, either as singlets with antiparallel spin or as triplets with parallel spin. The ratio between the two states depends on the orientation of the radical pair in the magnetic field and can thus mediate magnetic directions. The eye was suggested as the site of the receptive processes, because here light is available and, because of its more or less round form, receptors, aligned perpendicularly to its surface in ordered arrays, are covering all spatial directions. This would lead to a characteristic activation pattern on the retina that is centrally symmetric to the vector of the magnetic field; it can be spatially interpreted and thus indicate magnetic directions. As a receptor molecule, the authors suggested cryptochrome, a flavoprotein, because this is the only protein known in animals where absorption of photons leads to the formation of radical pairs [15,16].

This model is in agreement with the known characteristics of the avian magnetic compass. Because the singlet/triplet ratio depends on the course of the field lines, but not on their polarity, it results in an inclination compass, as found in birds. The ‘functional window’ and its flexibility can also be explained: the activation pattern also depends on magnetic intensity; birds that experience a sudden change in intensity are faced with a novel pattern which might be confusing at first. The pattern retains its central symmetry to the magnetic vector, however, so that birds can learn to interpret it after a while. Cryptochrome, the suggested magneto-receptor molecule, absorbs short-wavelength light from UV to about 560 nm (e.g. [17,18]) and is thus in agreement with the behavioural findings on the light dependency of compass orientation in birds (see also [19]).

Specific experiments support this model. Subjecting birds to radiofrequency fields, a diagnostic tool for radical pair processes [20], results in disorientation (figure 1*c*). This applies to migrants during migratory orientation [21–24] as well as to directionally trained non-migrants such as Domestic Chickens [25] and Zebra Finches [26]. The Larmor frequency, that is, the frequency of the electron, proved most effective and disrupts orientation at very low intensities [27]; this has been independently confirmed by [23,28]. Reports of the opposite [24] are inconclusive because of methodological short-comings (use of metallic test cages that not only shield but also reflect and distort radiofrequency fields so that the conditions become undefined).

### 2.2. Cryptochromes in the avian eyes

The chromophore of cryptochrome is flavin (FAD), which undergoes a redox cycle: photon absorption reduces FAD to the semiquinone FADH°, forming a first radical pair with tryptophan. In a next step, FADH° can be further photo-reduced to the fully reduced form FADH^−^, which is then re-oxidized independently of light. During this process, a second radical pair is formed (e.g. [18]). Behavioural experiments in flickering light and an alternatively pulsed magnetic field indicate that the receptive process itself does not require light, suggesting that, in contrast to cryptochrome-controlled responses in plants (e.g. [29]), the second radical pair formed during re-oxidation is the one crucial for avian magnetoreception. Light is required, however, to provide the fully reduced form FADH^−^ to be re-oxidized [30]. Details of this process are still not entirely clear (e.g. [31–36] and others).

Four types of cryptochromes—Cry1a, Cry1b, Cry2 and Cry4—have been found in the eyes of birds. Cryptochrome 1 was first described by Haque and colleagues [37] based on mRNA expression in the photoreceptor layer and the ganglion cell layer (see also [38]); Möller and colleagues [39] identified two splice products of the *Cry1* gene, Cry1a and Cry1b, with different C-termini. In an immuno-histochemical study using a specific antiserum, Nießner and colleagues [40] located Cry1a in the UVS/VS (SWS1) cones (ultraviolet/violet cones) in chickens and robins, with immuno-electron microscopy showing it positioned at the discs in the outer segment together with UV-opsin. An *in vivo* study showed that, in contrast to what Kutta and colleagues [41] assume, it is directly activated by light of the wavelengths that are absorbed by flavin [19]. The UVS/VS cones have clear oil droplets that allow all wavelengths of light to pass [42]; they are present all across the retina so that they can give rise to the activation pattern proposed by Ritz and colleagues [15] and mediate magnetic directions.

Cry1b was located by immuno-histochemistry in the cytosol of ganglion cells, displaced ganglion cells and also in the inner segments of the photoreceptors [43–45], free as well as bound to membranes. In night-migrating birds, its expression varies with season and was much stronger during the migratory season when the birds were active during the night [45,46]. A role in magnetoreception has been suggested [44], but, because the use of a magnetic compass is also shared by non-migrants and hence appears to be a general ability of birds, the seasonal changes seems to suggest another role, possibly involving the shift from diurnal activity to nocturnal *Zugunruhe*.

Cryptochrome 2 was described by Bailey and colleagues [47], who identified it by its mRNA in a number of organs, among them the pineal and, in the eyes, in the photoreceptors and ganglion cells (see also [38]). It includes a sequence that suggests its location in the cell nucleus [39,43], which points to a possible role as a clock protein (e.g. [48]).

Cryptochrome 4 was described in avian eyes by Watari and colleagues [49] using mRNA expression and immuno-histochemistry. With specific antisera, Günther and colleagues [50] could narrow down the location of Cry4 to the outer segments of the LWS (longwave-sensitive) single cones and of the double cones. It has recently been speculated that Cry4 may be involved in magnetoreception (e.g. [50,51]). In particular the location within the double cones prompted the suggestion of such a role of Cry4, because here the input of two adjacent receptors with the magneto-receptive molecules oriented in different directions could be compared to overcome problems by different light intensities and polarization [50,52]. However, the principal cone is associated with an oil droplet that acts as a cut-off filter absorbing short wavelengths [42] and thus most of the wavelengths needed for cryptochrome photo-reduction. This, together with the gap junctions between the two cones, would interfere with a comparison. Hence such a role of Cry4 in the double cones in magnetoreception seems problematic. The Cry4 in the LWS single cones seems even less suitable for magnetoreception, because these cones contain a red oil droplet that transmits only long wavelengths that are not absorbed by cryptochrome.

Qin and colleagues [53], based on genome-wide screenings, suggested an iron–sulfur protein polymer, MagR, with an intrinsic magnetic moment, in combination with Cry4, as a magnetic sensor that combines ferrimagnetic and light-dependent features. This complex was found by antibodies to be most highly expressed in the retinal ganglion cell layer, and the inner and outer nuclear layer. Yet the assumptions about the magnetic moment—it appears to be much weaker than assumed—and the immuno-histological studies have been criticized (see [54]), and it is still unclear whether such a combined sensor is involved in magnetoreception.

Altogether, the presently available evidence indicates Cry1a as the most likely receptor molecule for sensing directions. The observation that its gene expression shows a diurnal rhythm [51] does not speak against such a role, because it only indicates a rhythmic production in the inner segment, from where it has to be transported into the outer segment—a parallel case to vision where the production of opsins also shows a diurnal pattern (e.g. [55,56]).

### 2.3. Processing directional information in the brain

Any activation pattern caused by the magnetic field in the retina must be transmitted by the optic nerve to the brain to be processed (see [57,58]). How singlet/triplet ratios could be transformed into a sensory signal for transmission to the brain is not yet known.

Behavioural tests showed that, in adult birds, the magnetic compass is lateralized in favour of the right eye, which means that the respective information is processed predominantly in the left hemisphere of the brain [59–62]. Migrants could use both eyes during their first autumn migration [63–65]; the lateralization was found to develop between the first autumn and the first spring migration [65]. In the beginning, it proved rather flexible, as it could be undone by covering the right eye for 1½ h ([66]; see also [67], where that eye was covered for more than 1 h before the tests started). This suggests that the lateralization is initially caused by asymmetric inhibitory interactions between the two hemispheres; when the right eye was covered short-term synaptic plasticity returned the ability of the left eye and the right hemisphere to process magnetic directional information. Yet the lateralization in favour of the right eye returned once the right eye could be used again [66].

The centres in the brain where magnetic directional information is processed are still not entirely clear. Most probable candidates are areas associated with the visual system, yet it is still open whether the processing of magnetic information is an integrated part of vision or whether it is processed more or less independently as a sense of its own. The observation that covering the right eye could remove the lateralization only if meaningful magnetic information was available—light alone proved insufficient [66]—could be interpreted as suggesting the latter. An involvement of the thalamofugal pathway is suggested (e.g. [58,67,68]).

Electrophysiological responses to changes in magnetic directions were recorded from direction-sensitive cells in the nucleus of the basal optic root (nBOR), a part of the accessary optic system, and from the *stratum griseum et fibrosum superficiale* of the *tectum opticum* [69,70]. Yet recent studies failed to find magnetic field-induced activity in the *tectum opticum* [71,72].

Using neural activity markers, a part of the Wulst, cluster N, was identified as an area with considerable neural activity during migratory behaviour in night-migrating passerines when they had to rely on their magnetic compass [73]. A follow-up study showed activity in cluster N during nocturnal activity, but not during the day, with a certain lateralization in favour of the right hemisphere. This was discussed in connection with night vision, but also as possibly being associated with processing magnetic directional information, although a difference in neuronal activity between a near-zero magnetic field and a changing magnetic field could not be observed [74]. By neuronal tracing, cluster N was found to be connected with the retinal neurons via the visual thalamus by the thalamofugal pathway [58]. Lesioning cluster N led to disorientation [75]. However, when a day- and night-migrating passerine species, the Meadow Pipit *Anthus pratensis*, was tested, cluster N did not show enhanced activity during daytime migration [75], so that it remains open whether cluster N is indeed involved in magnetoreception or whether it controls other aspects of nocturnal migratory activity (see [72,74]). Studies comparing the activity of neuronal markers in birds subjected to a static magnetic field and in birds subjected to a rotating magnetic field indicated a certain increase in a number of brain areas, with the most pronounced one in the dorsomedial rostral hippocampus and some effect in a part of the hyperpallium [76].

Altogether, there are still a number of open questions about how a sensory signal is formed by the radical pair process, how the magnetic signal is separated from the visual information (see [77] for discussion) and about the brain areas where magnetic directional information is processed.

## 3. Magnetic ‘map’ components: magnetite-based receptors?

Because of their spatial distribution, magnetic intensity, declination and inclination could serve as components of the navigational ‘map’. Here, rather small differences must be recorded. The response of homing pigeons to natural fluctuations of the geomagnetic field suggests a sensitivity in the range of about 20 nT (nanotesla) [78]. To use declination and inclination, birds would have to record minute angular differences; additionally, these parameters require non-magnetic reference directions—true (astronomical) North and gravity, respectively—which would complicate their use.

Magnetic parameters are only one component of a multi-modal, redundant navigational ‘map’ [79]. The ‘map’ is established by experience. All young birds are assumed to familiarize themselves with the regional distribution of the ‘map’ factors in their home region by early exploration and dispersal flights. Migrants additionally acquire the respective knowledge on the ‘map’ factors for their extended journeys during their first migration, which is controlled by an innate migration programme (e.g. [80,81]); during later migrations, they are able to navigate ([82,83]; for a review, see [84]). The ‘map’ is assumed to include all factors that prove suitable in the respective region, which may be different in different parts of the world (e.g. [79]).

### 3.1. Effects of a magnetic pulse indicating receptors based on magnetic material

Theoretical considerations led to a number of hypotheses proposing magnetoreception by permanently magnetic particles (e.g. [85–87]). Birds indeed have a second type of magnetoreceptor apparently based on magnetite, a magnetic material of biogenic origin. Depending on particle size, magnetite has different magnetic properties: in larger particles, the magnetic moments tend to cancel each other; if the particles are sufficiently small—in the range between 0.04 and 0.12 µm—they consist of single domains with a permanent magnetic moment. Even smaller particles are superparamagnetic without stable magnetic moments, but their moments align in an external magnetic field.

An indicator for the involvement of magnetic material is the response to treatments with a strong magnetic pulse—strong enough to alter the magnetization of single-domain magnetite. Caged passerine migrants treated with a brief 0.5 T pulse showed a marked deviation from their migratory direction [88,89], with the size and the direction of this deflection depending on how the pulse was applied [90,91]. This suggests that the pulse did not silence the putative receptors altogether, but caused them to provide the birds with false information. The response to the pulse was restricted to experienced migrants that navigate towards an already familiar goal, whereas young birds on their first migration that fly innate courses were not affected [92]. This finding and the observation that homing pigeons treated with such a pulse deviated from untreated controls at some (but not all) sites in greater distances from home [93,94] indicate that the pulse affects a receptor that provides birds with a magnetic component of the navigational ‘map’ (see also [95]). Apparently, the pulse changes the course to be pursued, while the magnetic inclination compass remains unaffected [92,96]. This is also supported by later studies where migrants were subjected to magnetically simulated displacements (see below).

The effect of the pulse on the navigational system of migrants is short-lived, however: the deflection lasted only about 2–3 days; after this, the birds underwent a phase of disorientation, and about 10 days after the pulse treatment and later they again headed in their normal migratory direction [88]. These observations with caged migrants have a parallel in free-flying migrants: migrating birds caught at a stop-over site were treated with a pulse, released, and their departure directions were radio-tracked. Here, too, young birds on their first migration proved unaffected; adult migrants that departed within 10 days after pulse treatment were random, those that departed after 10 days were oriented in their normal migratory direction [97].

The relative short duration of the pulse effect—recovery of normal migratory orientation within about 10 days—could be an indication for the size of the magnetite particles involved. If the magnetization of single-domain particles was altered by the pulse, a new magnetization would be just as stable as the original one, and a complete exchange of the magnetic particles within a time span of just 10 days seems rather unlikely. Clusters of superparamagnetic particles, on the other hand, would be disrupted by the pulse, but could later rearrange themselves [98]. The observation that the pulse effect was not modified by an applied biasing field [99] could also by interpreted in favour of an involvement of superparamagnetic particles.

### 3.2. The location of magnetite-based receptors

The location and the structure of the magnetite-based receptors are still not entirely clear. After a number of studies had reported magnetic material in various places in the birds' head, attention focused on tiny iron-containing particles in the upper beak of pigeons. Electron-optical, magnetic remanence and micro-XANES (microscopic X-ray absorption near-edge structure) measurements identified clusters of superparamagnetic material [100–103], consisting of magnetite (Fe(II)Fe(III)_2_O_4_) and maghemite (Fe(III)_2_O_3_) [104]. Similar structures were also found in two species of migratory passerines and domestic chickens [104] so that they seemed to be a general feature of birds. Fleißner and colleagues [105,106] described specific subcellular structures of magnetite and maghemite in the dendrites of the ophthalmic branch, V1, of the trigeminal nerve. A theoretical analysis ascertained that the described structures could indeed provide the required magnetic information [107], while another analysis was not so sure [108].

The location of magnetoreceptors in the skin of the upper beak was in agreement with the observation that anaesthesia of the skin of the upper beak with a local anaesthetic suppressed the effect of the pulse [109]. Also, displaced homing pigeons with their beak anaesthetized were no longer confused in a strong magnetic anomaly; they left the site more rapidly, probably orienting by non-magnetic cues [110].

Another phenomenon associated with magnetoreceptors in the upper beak is a certain behaviour that occurs under unnatural light conditions where the normal inclination compass appears to be disrupted, such as total darkness, intense near-monochromatic light, or when yellow light is added to short-wavelength light. In these situations, migratory birds show so-called ‘fixed direction responses’ (figure 1*e*), that is, they prefer directions that are different from their normal migratory direction, do not reverse between autumn and spring and turned out to be polar responses to the magnetic field (figure 1*f*). Anaesthesia of the skin of the upper beak abolished these responses and led to disoriented behaviour (figure 1*h*). Hence the ‘fixed direction responses’ were attributed to the magnetite-based receptors located there; they are interpreted as possibly reflecting an ancient mechanism before the present inclination compass was developed (e.g. [2,111]).

Together, these findings supported magnetite-based receptors in the skin of the upper beak of birds. However, in 2010, Keays and colleagues [112] declared the iron-containing cells in the beak described by Fleißner and colleagues [105,106] to be macrophages. Also, in contrast to the authors mentioned above, they failed to find magnetite in the upper beak; yet, they used the spinning field method [113], which works well for single domains, but not for superparamagnetic particles. The disruptive effect of anaesthetizing the skin of the upper beak was also questioned, as a recent study seemed to show that, in spite of anaesthesia, changing magnetic fields led to magnetically induced activity in parts of the trigeminal brainstem [114]. This study, however, suffers from severe methodological short-comings: a type of anaesthetic, a spray, was used that differed from the injection solution used in the other studies (e.g. [2,109,111]). The effect of the spray, according to the product information sheet, lasts only 15–20 min, whereas the magnetic treatment was applied for 90 min. And the applied stimuli were irregularly changing and in part unnaturally strong, up to more than twice the intensity of the local geomagnetic field, while all the other studies with the anaesthetic took place in the natural geomagnetic field. Engels and colleagues [114] assume that the previous observed effect of local anaesthesia was unspecific. This is highly unlikely, however, because the treatment had no effect when birds could use their inclination compass (figure 1*d*; e.g. [2,111]), and migratory birds that had been subjected to a pulse showed oriented behaviour in their migratory direction when their beak was anaesthetized [109].

Additionally, magnetic material was reported from the otoliths of pigeons, particularly in the *lagena*, and discussed in connection with magneto-sensitivity [115]; iron-rich corpuscles were also found in hair cells in the *cochlea* of pigeons [116]. Yet their possible function as magnetoreceptors was questioned as they seemed unsuitable to provide magnetic information [117–119]. A reported behavioural response—slower homing of *lagena*-extirpated pigeons over a short distance [120]—is in contrast with an earlier, more extended study that had found no difference between *cochleae* and *lagenae*-extirpated pigeons and untreated control birds [121].

Presently, the specific location of the magnetite-based magnetoreceptors is still unclear. The observation that magnetic stimuli had been found to be transmitted by the ophthalmic nerve (see below) suggests that the receptors lie in the area innervated by this nerve, and here the region of the upper beak, where superparamagnetic magnetite had been described, seemed a likely candidate. Also the behavioural findings with the local anaesthesia supported receptors in that location. But although a recent study again searched very intensively in that area for potential magnetite-based receptors, it failed to identify any structure that would qualify [114].

### 3.3. Processing information from the magnetite-based receptors

In the mid-1980s, electrophysiological studies revealed responses to changes in the magnetic field recorded from the ophthalmic nerve of a passerine migrant, with about 15–20% of the spontaneously active units responding [122,123]. These responses continued when the direction of the magnetic field was held constant, indicating that the information concerned magnetic intensity [124].

Blocking the trigeminal nerve resulted in behavioural responses that indicate the transmission of magnetic intensity as navigational information. Treating the ophthalmic nerve with an anaesthetic suppressed the effect of the pulse in migrants: the birds continued in their migratory direction [125]. Birds—pigeons and ducks—were successfully conditioned to respond to an artificial local magnetic anomaly; when the upper beak was anaesthetized or the trigeminal nerve was sectioned, discrimination failed [126,127].

Most interesting are studies where migrants were displaced, either in reality or virtually by simulating the magnetic field conditions of a distant region. The birds compensated for the displacement and changed their course accordingly [128,129]; they could no longer do so when the trigeminal nerve was sectioned [130,131]. These findings clearly show that the magnetic information transmitted by the trigeminal nerve is used for navigation over longer distances.

In some experiments in Italy, sectioning the ophthalmic nerve did not affect the orientation behaviour of displaced homing pigeons (e.g. [132,133]). This might reflect the redundant nature of the navigational ‘map’: apparently, magnetic factors could be replaced by other, non-magnetic ones. The response of pigeons to magnetic anomalies (e.g. [110,134,135]) indicates that they normally consult magnetic information when available.

Units in the trigeminal ganglion responded in a similar way to those in the trigeminal nerve [136]. Later studies using immediate gene expression markers, ZENK, also revealed activity induced by magnetic stimuli in the trigeminal brainstem complex of passerine species and pigeons, in particular in the principal trigeminal sensory nucleus in the ascending tract and in the spinal trigeminal sensory nuclei [68,72,137]. This is in agreement with the behavioural data of blocking the trigeminal nerve mentioned above.

A study using cFos found magnetically induced activity in several parts of the brain of pigeons, among them the posterior vestibular nuclei, dorsal *thalamus*, *hippocampus* and the *hyperpallium*, which in part was attributed to receptors in the *lagena*, since the activity was reduced when the *lagena* was lesioned [138]. Electrophysiological recordings from single units in the vestibular nuclei produced responses to direction, intensity and polarity of the magnetic field [139]. The authors speculate that this may be the neural basis for a magnetic sense for navigation; yet a behavioural response to the magnetic field associated with the inner ear or the stato-acoustic nerve remains unknown.

## 4. Outlook

At present, the findings indicate that birds sense magnetic directions by radical pair processes in the eye, with the information mediated by the optic nerve, and probably magnetic intensity as a component of the navigational map by magnetite-based receptors in the region innervated by the ophthalmic branch of the trigeminal nerve. A possible role of magnetite particles in the inner ear is unclear.

Despite many successful studies in the last two decades, there are still a number of questions open, and there are several contradicting findings that have to be resolved. The primary processes of detecting directions—if they follow the radical pair model—appear to be largely understood, but how and where this information is transmitted and finally processed is still open. The sensing of a magnetic ‘map’ component is characterized by the striking discrepancy between the transmission of magnetic information in the ophthalmic branch of the trigeminal nerve being well documented by electrophysiology, neuronal activity markers and behavioural data, and the fact that any receptive structures in the area innervated by this nerve could not yet be securely identified.

The magnetic stimuli used to identify structures processing magnetic information are not entirely unproblematic, because they have to be completely unnatural. They normally include rapid changes in direction as well as in magnetic intensity at an order of magnitude that never occurs in nature. The geomagnetic field is more or less stable and never undergoes sudden changes; temporal and spatial variations, as they are caused by rock magnetization in the ground, daily variations and magnetic storms, are minute. In contrast to most other sensory systems that evolved to detect *changes* in the environment, the magnetic compass system is built to extract information from a situation that never changes, and the receptors of the ‘map’ system must have evolved to detect very small and subtle gradual changes. We can only hope that in spite of the highly unnatural stimuli used the observed responses reflect the sensory apparatus realistically.

We are only just beginning to understand the processing of magnetic information in the brain. For directions as well as intensity, a few regions are indicated, but where the more complex processes combining magnetic information with other relevant information for navigation take place is largely unknown, e.g. where directional information from the magnetic field is combined with directional information from the Sun and the stars, and where the magnetic component of the navigational map is integrated with the other components to allow the birds to determine their whereabouts and the compass course to the desired goal. Mouritsen and colleagues [140] discuss several possibilities in some detail. One might consider the *hippocampus* as a most likely candidate for integrating all these types of information—however, displacement experiments with hippocampal-ablated pigeons showed that these birds departed homeward oriented like the control birds (e.g. [141]), i.e. navigation from greater distances could still take place. Further research will hopefully lead to a more complete picture of where in the brain magnetic information is processed and combined with other information for navigational processes.

Finally, a note of caution: the summary on magnetoreception reported here applies to birds only—in particular, where the compass is concerned, it appears to reflect a special development of the birds. Other vertebrates seem to have different ways of sensing magnetic directions. Fish and mammals have a polarity compass [142–144], amphibians and reptiles also have an inclination compass [145,146], but that of amphibians shows a wavelength dependency that is different from that of birds [147], and that of marine turtles does not require light (e.g. [148]). Possible magnetic ‘map’ components have been studied in only a few other vertebrates, with marine turtles being the group that has been most thoroughly studied by far. Here, Lohmann and colleagues [149], in a pioneering study, tested Green Sea–turtles, *Chelonia mydas*, under magnetic conditions at two locations about 340 km north and south of the test site; they found that the turtles compensated for this magnetically simulated displacement, indicating that, for them, too, magnetic factors are an important component of long-distance navigation. The sensory mechanisms involved in obtaining potential magnetic ‘map’ information in non-avian vertebrates are still unknown.

## Data accessibility

This article has no additional data.

## References

[1] Wiltschko R, Wiltschko W 2014 Sensing magnetic direction in birds: radical pair processes involving cryptochrome. Biosensors 4, 221–242. (10.3390/bios4030221) 25587420 PMC4264356

[2] Wiltschko R, Stapput K, Thalau P, Wiltschko W 2010 Directional orientation of birds by the magnetic field under different light conditions. J. R. Soc. Interface 7, S163–S177. (10.1098/rsif.2009.0367.focus) 19864263 PMC2843996

[3] Wiltschko W, Wiltschko R 1972 The magnetic compass of European robins. Science 176, 62–64. (10.1126/science.176.4030.62) 17784420

[4] Beason RC 1989 Use of an inclination compass during migratory orientation by the bobolink (*Dolichonyx oryzivorus*). J. Ornithol. 128, 317–324. (10.1007/BF01640301)

[5] Wiltschko W 1978 Further analysis of the magnetic compass of migratory birds. In Animal migration, navigation, and homing (eds Schmidt-Koenig K, Keeton WT), pp. 302–310. Berlin, Germany: Springer.

[6] Wiltschko W, Stapput K., Thalau P, Wiltschko R 2006 Avian magnetic compass: fast adjustment to intensities outside the normal functional window. Naturwissenschaften 93, 300–304. (10.1007/s00114-006-0102-5) 16586120

[7] Winklhofer M, Dylda E, Thalau P, Wiltschko W, Wiltschko R 2013 Avian magnetic compass can be tuned to anomalously low magnetic intensities. Proc. R. Soc. B 280, 20130850 (10.1098/rspb.2013.0853) PMC3774234 23720547

[8] Wiltschko W, Munro U, Ford H, Wiltschko R 1993 Red light disrupts magnetic orientation of migratory birds. Nature 364, 525–527. (10.1038/364525a0)

[9] Wiltschko W, Wiltschko R 1995 Migratory orientation of European robins is affected by the wavelength of light as well as by a magnetic pulse. J. Comp. Physiol. A 177, 363–369. (10.1007/BF00192425)

[10] Rappl R, Wiltschko R, Weindler P, Berthold P, Wiltschko W 2000 Orientation behavior of garden warblers (*Sylvia borin*) under monochromatic light of various wavelength. Auk 117, 256–269. (10.1642/0004-8038(2000)117[0256:OBOGWS]2.0.CO;2)

[11] Muheim R, Bäckman J, Åkesson S 2002 Magnetic compass orientation in European robins is dependent on both wavelengths and intensity of light. J. Exp. Biol. 205, 3845–3856. 12432008 10.1242/jeb.205.24.3845

[12] Pinzon-Rodriguez A, Muheim R 2017 Zebra finches have a light-dependent magnetic compass similar to migratory birds. J. Exp. Biol. 220, 1202–1299. (10.1242/jeb.148098) 28356366

[13] Åkesson S, Morin J, Muheim R, Ottoson U 2001 Avian orientation at steep angles of inclination: experiments with migratory white-crowned sparrows at the magnetic North Pole. Proc. R. Soc. Lond. B 268, 1907–1913. (10.1098/rspb.2001.1736) PMC1088826 11564346

[14] Schwarze S, Steenken F, Thiele N, Kobolkov D, Lehfeld N, Dreyer D, Schneider NL, Mouritsen H 2016 Migratory blackcaps can use their magnetic compass at 5 degrees inclination, but are completely random at 0 degrees inclination. Sci. Rep. 6, 33805 (10.1038/srep33805) 27667569 PMC5036058

[15] Ritz T, Adem S, Schulten K 2000 A model for photoreceptor-based magnetoreception in birds. Biophys. J. 78, 707–718. (10.1016/S0006-3495(00)76629-X) 10653784 PMC1300674

[16] Xu BM, Zou J, Li H, Li JG, Shao B 2014 Effects of radio frequency field on the radical pair magnetoreception model. Phys. Rev. E 90, 042711 (10.1103/physreve.90.042711) 25375527

[17] Chaves I et al. 2011 The cryptochromes: blue light photoreceptors in plants and animals. Annu. Rev. Plant Biol. 62, 335–364. (10.1146/annurev-arplant-042110-103759) 21526969

[18] Müller P, Ahmad M 2011 Light-activated cryptochrome reacts with molecular oxygen to form a flavin-superoxide radical pair consistent with magnetoreception. J. Biol. Chem. 286, 21 033–21 040. (10.1074/jbc.M111.228940) PMC3122164 21467031

[19] Nießner C, Denzau S, Stapput K, Ahmad M, Peichl L, Wiltschko W, Wiltschko R 2013 Magnetoreception: activated cryptochrome 1a concurs with magnetic orientation in birds. J. R. Soc. Interface 10, 20130630 (10.1098/rsif.2013.0638) 23966619 PMC3785833

[20] Henbest KB, Kurokova P, Rodgers CT, Hore PJ, Timmel CR 2004 Radio frequency magnetic field effects on a radial recombination reaction: a diagnostic test for the radical pair mechanism. J. Am. Chem. Soc. 126, 8102–8103. (10.1021/ja048220q) 15225036

[21] Ritz T, Thalau P, Philllips JB, Wiltschko R, Wiltschko W 2004 Resonance effects indicate a radical-pair mechanism for avian magnetic compass. Nature 429, 177–180. (10.1038/nature02534) 15141211

[22] Thalau P, Ritz T, Stapput K, Wiltschko R, Wiltschko W 2005 Magnetic compass orientation of migratory birds in the presence of a 1.315 MHz oscillating field. Naturwissenschaften 92, 86–90. (10.1007/s00114-004-0595-8) 15614508

[23] Kavokin K, Chernetsov N, Pakomov A, Bojarinova J, Kobylkov D, Namozov B 2014 Magnetic orientation in garden warblers (*Sylvia borin*) under 1.4 MHz radiofrequency field. J. R. Soc. Interface 11, 20140451 (10.1098/rsif.2014.0451) 24942848 PMC4208380

[24] Schwarze S, Schneider NL, Reichl T, Dreyer D, Lefeldt N, Engels S, Baker N, Hore PJ, Mouritsen H 2016 Weak broadband electromagnetic fields are more disruptive to magnetic compass orientation in a night-migratory songbird (*Erithacus rubecula*) than strong narrow-band fields. Front. Behav. Neurosci. 10, 55 (10.3389/fnbeh.2016.00055) 27047356 PMC4801848

[25] Wiltschko W, Freire R, Munro U, Ritz T, Rogers L, Thalau P, Wiltschko R 2007 The magnetic compass of domestic chickens, *Gallus gallus*. J. Exp. Biol. 210, 2300–2310 (10.1242/jeb.004853) 17575035

[26] Keary N, Ruploh T, Voss J, Thalau P, Wiltschko R, Wiltschko W, Bischof HJ 2009 Oscillating magnetic field disrupts magnetic orientation in zebra finches, *Taeniopygia guttata**.* Front. Zool. 6, 25 (10.1186/1742-9994-6-25) 19852792 PMC2774300

[27] Ritz T, Wiltschko R, Hore PJ, Rodgers CT, Stapput K, Thalau P, Timmel CR, Wiltschko W 2009 Magnetic compass of birds is based on a molecule with optimal directional sensitivity. Biophys. J. 96, 3451–3457. (10.1016/j.bpj.2008.11.072) 19383488 PMC2718301

[28] Pakhomov A, Bojarinova J, Cherbunin R, Chetverikova R, Gigoryev PS, Kavokin K, Kobylkov D, Lubkoskaja R, Cherbakov N 2017 Very weak oscillating magnetic field disrupts the magnetic compass of songbird migrants. J. R. Soc. Interface 14, 20170364 (10.1098/rsif.2017.0364) 28794163 PMC5582129

[29] Bouly JP et al. 2007 Cryptochrome blue light photoreceptors are activated through interconversion of flavin redox states. J. Biol. Chem. 282, 9383–9391. (10.1074/jbc.M609842200) 17237227

[30] Wiltschko R, Ahmad M, Nießner C, Gehring D, Wiltschko W 2016 Light-dependent magnetoreception in birds: the crucial step occurs in the dark. J. R. Soc. Interface 13, 20151010 (10.1098/rsif.2015.1010) 27146685 PMC4892254

[31] Hogben HJ, Efimova O, Wagner-Rundell N, Timmel CR, Hore PJ 2009 Possible involvement of superoxide and dioxygen with cryptochrome in avian magnetoreception: origin of Zeeman resonances observed by *in vivo* EPR spectroscopy. Chem. Phys. Lett. 480, 118–132. (10.1016/j.cplett.2009.08.051)

[32] Hogben HJ, Biskup T, Hore PJ 2012 Entanglement and sources of magnetic anisotropy in radical pair-based avian magnetoreceptors. Phys. Rev. Lett. 109, 220501 (10.1103/PhysRevLett.109.220501) 23368109

[33] Lee AA, Lau JCS, Hogben HJ, Biskup T, Kattnig DR, Hore PJ 2014 Alternative radical pairs for cryptochrome-based magnetoreception. J. R. Soc. Interface 11, 20131063 (10.1098/rsif.2013.1063) 24671932 PMC4006233

[34] Worster S, Kattnig DR, Hore PJ 2016 Spin relaxation of radicals in cryptochrome and its role in avian magnetoreception. J. Chem. Phys. 145, 035104 (10.1063/1.4958624) 27448908

[35] Kattnig D 2017 Radical-pair based magnetic reception amplified by radical scavenging: resilience to spin relaxation. J. Phys. Chem. B. 121, 10 215–10 227. (10.1021/acs.jpcb.7b07672) 29028342

[36] Nielsen C, Kattnig DR, Sjulstok E, Hore PJ, Solov'yov IA 2017 Ascorbic acid may not be involved in cryptochrome-based magnetoreception. J. R. Soc. Interface 14, 2017065 (10.1098/rsif.2017.0657) PMC5746572 29263128

[37] Haque R, Chaurasia SS, Wessel JH III, Iuvone PM 2002 Dual regulation of cryptochrome I mRNA expression in chicken retina by light and circadian oscillations. Neuroreport 13, 2247–2251. (10.1097/00001756-200212030-00016) 12488805

[38] Fu Z, Inaba M, Noguchi T, Kato H 2002 Molecular cloning and circadian regulation of cryptochrome genes in Japanese quails (*Cotunix coturnix japonica*). J. Biol. Rhythms 17, 14–27. (10.1177/074873002129002302) 11837944

[39] Möller A, Sagasser S, Wiltschko W, Schierwater B 2004 Retinal cryptochrome in a migratory passerine bird: a possible transducer for the avian magnetic compass. Naturwissenschaften 91, 585–588. (10.1007/s00114-004-0578-9) 15551029

[40] Nießner C, Denzau S, Gross JC, Peichl L, Bischof HJ, Fleißner G, Wiltschko W, Wiltschko R 2011 Avian ultraviolet/violet cones identified as probable magnetoreceptors. PLoS ONE 6, 20091 (10.1371/journal.pone.0020091) PMC3102070 21647441

[41] Kutta RJ, Archipowa N, Johannissen LO, Jones AR, Scrutton NS 2017 Vertebrate cryptochromes are vestigial flavoproteins. Sci. Rep. 7, 44906 (10.1038/srep44906) 28317918 PMC5357904

[42] Hart NS 2001 The visual ecology of avian photoreceptors. Prog. Retin. Eye Res. 20, 675–703. (10.1016/S1350-9462(01)00009-X) 11470455

[43] Mouritsen H, Janssen-Bienhold U, Liedvogel M, Feenders G, Stalleicken J, Dirks P, Weiler R 2004 Cryptochrome and activity markers co-localize in bird retina during magnetic orientation. Proc. Natl Acad. Sci. USA 101, 14 294–14 299. (10.1073/pnas.0405968101) 15381765 PMC521149

[44] Bolte P et al. 2016 Localisation of the putative magnetoreceptive protein cryptochrome 1b in the retinae of migratory birds and homing pigeons. PLoS ONE 11, e0147819 (10.1371/journal.pone.0147819) 26953791 PMC4783096

[45] Nießner C, Gross JC, Denzau S, Peichl L, Fleißner G, Wiltschko R, Wiltschko W 2016 Seasonally changing cryptochrome 1b expression in the retinal ganglion cells of a migrating passerine bird. PLoS ONE 11, e0150377 (10.1371/journal.pone.0150377) 26953690 PMC4783048

[46] Fusani L, Bertolucci C, Frigato E, Foa A 2014 Cryptochrome expression in the eye of migratory birds depends on their migratory status. J. Exp. Biol. 217, 918–923. (10.1242/jeb.096479) 24622895

[47] Bailey MJ, Chong NW, Xiong J, Cassone VM 2002 Chickens’ Cry2: molecular analysis of an avian cryptochrome in retinal and pineal photoreceptors. FEBS Lett. 513, 169–174. (10.1016/S0014-5793(02)02276-7) 11904144

[48] Sancar A 2003 Structure and function of DNA photolyase and cryptochrome blue-light photoreceptors. Chem. Rev. 103, 2203–2237. (10.1021/cr0204348) 12797829

[49] Watari R, Yamaguch C, Zemba W, Kubo Y, Okano K 2012 Light-dependent structural change in chicken retinal cryptochrome 4. J. Biol. Biochem. 287, 42 634–42 641. (10.1074/jbc.m112.395731) PMC3522264 23095750

[50] Günther A, Einwich A, Sjulstok E, Feederle R, Bolte P, Koch KW, Solv'yov AI, Mouritsen H 2018 Double-cone localization and seasonal expression pattern suggest a role in magnetoreception for European robin cryptochrome 4. Curr. Biol. 28, 211–223. (10.1016/j.cub.2017.12.003) 29307554

[51] Pinzon-Rodriguez A, Bensch S, Muheim R 2018 Expression patterns of cryptochrome genes in avian retina suggest involvement of Cry4 in light-dependent magnetoreception. J. R. Soc. Interface 15, 20180058 (10.1098/rsif.2018.0058) 29593090 PMC5908540

[52] Worster S, Mouritsen H, Hore PJ 2017 A light-dependent magnetoreception mechanism insensitive to light intensity and polarization. J. R. Soc. Interface 12, 20170405 (10.1098/rsif.2017.0405) PMC5636276 28878033

[53] Qin S et al. 2015 A magnetic protein biocompass. Nat. Mater. 15, 217–226. (10.1038/nmat4484) 26569474

[54] Nießner C, Winklhofer M 2017 Radical-pair-based magnetoreception in birds: radio-frequency experiments and the role of cryptochrome. J. Comp. Physiol. A 203, 499–507. (10.1007/s00359-017-1189-1) PMC5522499 28612234

[55] Pierce ME, Sheshberadaran H, Zhang Z, Fox LE, Applebury ML, Takahashi JS 1993 Circadian regulation of iodopsin gene expression in embryonic photoreceptors in retinal cell culture. Neuron 10, 579–584. (10.1016/0896-6273(93)90161-J) 8476610

[56] von Schantz M, Lucas RJ, Foster RG. 1999 Circadian oscillation of photo-pigment transcript levels in the mouse retina. Mol. Brain. Res. 72, 108–114. (10.1016/S0169-328X(99)00209-0) 10521605

[57] Heyers D, Manns M, Luksch H, Güntürkün O, Mouritsen H 2007 A visual pathway links brain structures active during magnetic compass orientation in migratory birds. PLoS ONE 2, e937 (10.1371/journal.pone.0000937) 17895978 PMC1976598

[58] Zapka M et al. 2009 Visual but not trigeminal mediation of magnetic compass information in a migratory bird. Nature 461, 1274–1278. (10.1038/nature08528) 19865170

[59] Wiltschko W, Traudt J, Güntürkün O, Prior H, Wiltschko R 2002 Lateralisation of magnetic compass orientation in a migratory bird. Nature 419, 467–470. (10.1038/nature00958) 12368853

[60] Wiltschko W, Munro U, Ford H, Wiltschko R 2003 Lateralisation of magnetic compass orientation in silvereyes, *Zosterops lateralis*. Aust. J. Zool. 51, 597–602. (10.1071/ZO03022)

[61] Rogers LJ, Munro U, Freire R, Wiltschko R, Wiltschko W 2008 Lateralized response of chicks to magnetic cues. Behav. Brain Res. 186, 66–71. (10.1016/j.bbr.2007.07.029) 17765981

[62] Wilzeck C, Wiltschko W, Güntürkün O, Wiltschko R, Prior H 2010 Lateralization of the magnetic compass orientation in pigeons. J. R. Soc. Interface 7, S235–S240. (10.1098/rsif.2009.0436.focus) 20053653 PMC2843993

[63] Hein CM, Zapka M, Heyers D, Kutzschbauch S, Schneider NL, Mouritsen H 2010 Night-migrating garden warblers can orient with their magnetic compass using the left, the right or both eyes. J. R. Soc. Interface 7, S227–S233. (10.1098/rsif.2009.0376.focus) 19889693 PMC2844002

[64] Hein CM, Engels S, Kishkinev D, Mouriten H 2011 Robins have a magnetic compass in both eyes. Nature 471, E11–E12. (10.1038/nature09875) 21455128

[65] Gehring D, Wiltschko W, Güntürkün O, Denzau S, Wiltschko R 2012 Development of lateralization of the magnetic compass in a migratory bird. Proc. R. Soc. B 279, 4230–4235. (10.1098/rspb.2012.1654) PMC3441093 22933375

[66] Gehring D, Güntürkün O, Wiltschko W, Wiltschko R 2017 Lateralization of the avian magnetic compass: analysis of its early plasticity. Symmetry 9, 77 (10.3390/sym9050077)

[67] Engels S, Hein MC, Lefeld N, Prior H, Mouritsen H 2012 Night-migratory songbirds possess a magnetic compass in both eyes. PLoS ONE 7, e43271 (10.1371/journal.pone.0043271) 22984416 PMC3440406

[68] Heyers D, Zapka M, Hoffmeister M, Wild JM, Mouritsen H 2010 Magnetic field changes activate the trigeminal brainstem complex in a migratory bird. Proc. Natl Acad. Sci. USA 107, 9394–9399. (10.1073/pnas.0907068107) 20439705 PMC2889125

[69] Semm P, Nohr D, Demaine C, Wiltschko W 1984 Neural basis of the magnetic compass: interactions of visual, magnetic and vestibular inputs in the pigeon's brain. J. Comp. Physiol. 155, 283–288. (10.1007/BF00610581)

[70] Semm P, Demaine C 1986 Neurophysiological properties of magnetic cells in the pigeon's visual system. J. Comp. Physiol. A 159, 619–625. (10.1007/BF00612035) 3806432

[71] Ramirez E, Marin G, Mpodozis M, Letelier JC 2014 Extracellular recordings reveal absence of magneto-sensitive units in the avian optic tectum. J. Comp. Physiol. A 200, 983–996. (10.1007/s00359-014-0947-6) PMC4237910 25281335

[72] Elbers D, Bulte M, Bairlein F, Mouritsen H, Heyers D 2017 Magnetic activation in the brain of the migratory northern wheatear (*Oenanthe oenanthe*). J. Comp. Physiol. A 203, 591–600. (10.1007/s00359-017-1167-7) 28361169

[73] Mouritsen H, Feenders G, Liedvogel M, Wada K, Jarvis ED 2005 Night-vision brain area in migratory songbirds. Proc. Natl Acad. Sci. USA 102, 8339–8344. (10.1073/pnas.0409575102) 15928090 PMC1149410

[74] Liedvogel M, Feenders G, Wada K, Troje NF, Jarvis ED, Mouritsen H 2007 Lateralized activation of cluster N in the brains of migratory songbirds. Eur. J. Neurosci. 25, 1166–1173. (10.1111/j.1460-9568.2007.05350.x) 17331212 PMC2475547

[75] Zapka M, Heyers D, Liedvogel M, Jarvia ED, Mouristen H 2010 Night-time neuronal activation of cluster N in a day- and night-migrating songbird. Eur. J. Neurosci. 32, 619–624 (10.1111/j.1460-9568.2010.07311.x) 20618826 PMC2924469

[76] Keary N, Bischof H 2012 Activation changes in zebra finch (*Taeniopygia guttata*) brain areas evoked by alterations of the Earth magnetic field. PLoS ONE 7, e38697 (10.1371/journal.pone.0038697) 22679515 PMC3367956

[77] Bischof HJ, Nießner C, Peichl L, Wiltschko R, Wiltschko W 2011 Avian UV/violet cones as magnetoreceptors: the problem of separating visual and magnetic information. Commun. Integr. Biol. 4, 713–716. (10.4161/cib.17338) 22446535 PMC3306339

[78] Schiffner I, Wiltschko R 2011 Temporal fluctuations of the geomagnetic field affect pigeons’ entire homing flight. J. Comp. Physiol. A 197, 765–772. (10.1007/s00359-011-0640-y) 21451981

[79] Walcott C 2005 Multi-modal orientation cues in homing pigeons. Integr. Comp. Biol. 45, 574–581. (10.1093/icb/45.3.574) 21676803

[80] Berthold P, Querner U 1981 Genetic basis of migratory behavior in European warblers. Science 212, 77–79. (10.1126/science.212.4490.77) 17747634

[81] Berthold P 1988 The control of migration in European warblers. In *Acta XIX Congr. Internat. Ornithol. Ottawa* (ed. H Ouellet), pp. 215–249 Ottawa, Canada: University of Ottawa Press.

[82] Perdeck AC 1958 Two types of orientation in migrating starlings, *Sturnus vulgaris* L., and chaffinches, *Fringilla coelebs* L. as revealed by displacement experiments. Ardea 46, 1–37. (10.5253/arde.v1i2.p1)

[83] Thorup K, Bisson IA, Bowlin MS, Holland RA, Wingfield JC, Ramenofsky M, Wikelski M 2007 Evidence for a navigational map stretching across the continental U.S. in migratory songbird. Proc. Natl Acad. Sci. USA 104, 18 115–18 119. (10.1073/pnas.0704734104) PMC2084305 17986618

[84] Wiltschko R 2017 Navigation. J. Comp. Physiol. A 203, 455–463. (10.1007/s00359-017-1160-1) 28289837

[85] Yorke ED 1979 A possible magnetic transducer in birds. J. Theor. Biol. 77, 101–105. (10.1016/0022-5193(79)90140-1) 449364

[86] Kirschvink JL, Gould JL 1981 Biogenic magnetite as a basis for magnetic field detection in animals. Biosystems 13, 181–201. (10.1016/0303-2647(81)90060-5) 7213948

[87] Shcherbakov VP, Winklhofer M 1999 The osmotic magnetometer: a new model for magnetite-based magnetoreceptors in animals. Eur. Biophys. J. 28, 380–392. (10.1007/s002490050222)

[88] Wiltschko W, Munro U, Beason RC, Ford H, Wiltschko R 1994 A magnetic pulse leads to a temporary deflection in the orientation of migratory birds. Experientia 50, 697–700. (10.1007/BF01952877)

[89] Wiltschko W, Munro U, Ford H, Wiltschko R 1998 Effect of a magnetic pulse on the orientation of silvereyes, *Zosterops l. lateralis*, during spring migration. J. Exp. Biol. 201, 3257–3261. (10.1071/zo03022) 9808838

[90] Beason RC, Dussourd N, Deutschlander MC 1995 Behavioral evidence for the use of magnetic material in magnetoreception by a migratory bird. J. Exp. Biol. 198, 141–146. 9317510 10.1242/jeb.198.1.141

[91] Holland RA 2010 Differential effects of magnetic pulses on the orientation of naturally migrating birds. J. R. Soc. Interface 7, 1617–1625. (10.1098/rsif.2010.0159) 20453067 PMC2988258

[92] Munro U, Munro JA, Phillips JB, Wiltschko R, Wiltschko W 1997 Evidence for a magnetite-based navigational ‘map’ in birds. Naturwissenschaften 84, 26 (10.1007/s001140050343)

[93] Beason RC, Wiltschko R, Wiltschko W 1997 Pigeon homing: effects of magnetic pulses on initial orientation. Auk 114, 405–415. (10.2307/4089242)

[94] Holland RA, Filannino C, Gagliardo A 2013 A magnetic pulse does not affect homing pigeon navigation: a GPS tracking experiment. J. Exp. Biol. 216, 2192–2200. (10.1242/jeb.083543) 23470658

[95] Mora CV, Bingman VP 2013 Detection of magnetic field intensity gradient by homing pigeons (*Columba livia*) in a novel ‘virtual magnetic map’ conditioning paradigm. PLoS ONE 8, e72869 (10.1371/journal.pone.0072869) 24039812 PMC3767695

[96] Wiltschko W, Munro U, Ford H, Wiltschko R 2006 Bird navigation: what type of information does the magnetite-based receptor provide? Proc. R. Soc. B 273, 2815–2850. (10.1098/rspb.2006.3651) PMC1664630 17015316

[97] Holland RA, Helm B 2013 A strong magnetic pulse affects the precision of departure of naturally migrating adult but not juvenile birds. J. R. Soc. Interface 10, 20121047 (10.1098/rsif.2012.1047) 23389901 PMC3627120

[98] Davila AF, Winklhofer M, Shcherbakov VP, Petersen N 2005 Magnetic pulse affects a putative magnetoreceptor mechanism. Biophys. J. 89, 56–63. (10.1529/biophysj.104.049346) 15863473 PMC1366555

[99] Wiltschko W, Munro U, Wiltschko R, Kirschvink J 2002 Magnetite-based magnetoreception in birds: the effect of a biasing field and a pulse on migratory behavior. J. Exp. Biol. 205, 3031–3037. 12200406 10.1242/jeb.205.19.3031

[100] Hanzlik M, Heunemann E, Holtkamp-Rötzler E, Winklhofer M, Petersen N, Fleißner G 2000 Superparamagnetic magnetite in the upper beak of homing pigeons. Biometals 13, 325–331. (10.1023/A:1009214526685) 11247039

[101] Williams MN, Wild JM 2001 Trigeminally innervated iron-containing structures in the beak of homing pigeons, and other birds. Brain Res. 889, 243–246. (10.1016/S0006-8993(00)03114-0) 11166712

[102] Winklhofer M, Holtlamp-Rötzler E, Hanzlik M, Fleißner G, Petersen N 2001 Clusters of superparamagnetic magnetite particles in the upper-beak skin of homing pigeons: evidence of a magnetoreceptor? Eur. J. Mineral. 13, 659–669. (10.1127/0935-1221/2001/0013-0659)

[103] Tian L, Xiao B, Lin W, Zhang S, Zhu R, Pan Y 2007 Testing for the presence of magnetite in the upper-beak skin of homing pigeons. Biometals 20, 197–203. (10.1007/s10534-006-9027-x) 16900396

[104] Falkenberg G, Fleißner G, Schuchart K, Kuehbacher M, Thalau P, Mouritsen H, Heyers D, Wellenreuther G, Fleißner G 2010 Avian magnetoreception: elaborate iron mineral containing dendrites in the upper beak seem to be a common feature in birds. PLoS ONE 5, e9231 (10.1371/journal.pone.0009231) 20169083 PMC2821931

[105] Fleißner G, Holtkamp-Rötzler E, Hanzlik M, Winklhofer M, Fleißner G, Petersen N, Wiltschko W 2003 Ultrastructural analysis of a putative magnetoreceptor in the beak of homing pigeons. J. Comp. Neurol. 458, 350–360. (10.1002/cne.10579) 12619070

[106] Fleißner G, Stahl B, Thalau P, Falkenberg G, Fleißner G 2007 A novel concept of Fe-mineral-based magnetoreception: histological and physicochemical data from the upper beak of homing pigeons. Naturwissenschaften 94, 631–642. (10.1007/s00114-007-0236-0) 17361399

[107] Solov'yov I.A, Greiner W 2007 Theoretical analysis of an iron mineral-based magnetoreceptor model in birds. Biophys. J. 93, 1493–1509. (10.1529/biophysj.107.105098) 17496012 PMC1948037

[108] Jandacka P, Alexa P, Pistora J, Trojkova J 2013 Hypothetical superparamagnetic magnetometer in a pigeon's upper beak probably does not work. Eur. Phys. J. E 36, 40 (10.1140/epje/i2013-13040-1) 23605568

[109] Wiltschko W, Munro U, Ford H, Wiltschko R 2009 Avian orientation: the pulse effect is mediated by the magnetite receptors in the upper beak. Proc. R. Soc. B 276, 2227–2232. (10.1098/rspb.2009.0050) PMC2677601 19324756

[110] Wiltschko R, Schiffner I, Fuhrmann P, Wiltschko W 2010 The role of the magnetite-based receptors in the beak in pigeon homing. Curr. Biol. 20, 1534–1538. (10.1016/j.cub.2010.06.073) 20691593

[111] Wiltschko R, Dehe L, Gehring D, Thalau P, Wiltschko W 2013 Interaction between the visual and the magnetoreception system: different effects of bichromatic light regimes on the directional behavior of migratory birds. J. Physiol. (Paris) 107, 137–146. (10.1016/j.jphysparis.2012.03.003) 22504660

[112] Treiber CD et al. 2012 Clusters of iron-rich cells in the upper beak of pigeons are macrophages not magnetosensitive neurons. Nature 484, 367–370. (10.1038/nature11046) 22495303

[113] Edelman NB et al. 2015 No evidence for intracellular magnetite in putative vertebrate magnetoreceptors identified by magnetic screening. Proc. Natl Acad. Sci. USA 112, 262–267. (10.1073/pnas.1407915112) 25535350 PMC4291630

[114] Engels S, Treiber CD, Salzer MC, Michalik A, Ushakova L, Keays DA, Mouritsen H, Heyers D 2018 Lidocaine is a nocebo treatment for trigeminally mediated magnetic orientation in birds. J. R. Soc. Interface 15, 20180124 (10.1098/rsif.2018.0124) 30089685 PMC6127160

[115] Harada Y, Tanguch M, Namatame H, Ilda A 2001 Magnetic materials in otoliths of bird and fish lagena and their function. Acta Otolaryngol. 121, 590–595. (10.1080/000164801316878872) 11583391

[116] Lauwers M et al. 2013 An iron-rich organelle in the cuticular plate of avian hair cells. Curr. Biol. 23, 924–929. (10.1016/j.cub.2013.04.025) 23623555

[117] Zhao Y, Huang YN, Shi L, Chen L 2009 Analysis of magnetic elements of otoliths of the macula lagena in homing pigeons with inductively coupled plasma mass spectrometry. Neurosci. Bull. 25, 101–108. (10.1007/s12264-009-0311-y) 19448683 PMC5552564

[118] Jandacka P, Burda H, Pistora J 2014 Magnetically induced behavior of ferritin corpuscles in avian ears: can cuticulosomes function as magnetosomes? J. R. Soc. Interface 12, 20141087 (10.1098/rsif.2014.1087) PMC4277103 25551148

[119] Malkemper EP, Ushakova L, Nimp S, Pichler P, Treiber CD, de Jonge M, Shaw J, Keays DA. 2019 No evidence for a magnetite-based magnetoreceptor in the lagena of pigeons. Curr. Biol. 29, R1–R15. (10.1016/j.cub.2018.11.032) 30620907

[120] Harada Y 2002 Experimental analysis of behavior of homing pigeons as a result of functional disorders of their lagena. Acta Otolaryngol. 122, 132–137. (10.1080/00016480252814126) 11936903

[121] Wallraff HG 1972 Homing of pigeons after extirpation of their cochleae and lagenae. Nat. New Biol. 236, 223–224. (10.1038/newbio236223a0) 4537002

[122] Beason RC 1986 Magnetic orientation and magnetically sensitive material in migratory birds. In Biophysical effects of steady magnetic fields (eds Maret G, Boccara N, Kiepenheuer J), pp. 167–172. Berlin, Germany: Springer.

[123] Beason RC, Semm P 1987 Magnetic responses of the trigeminal nerve system of the bobolink (*Dolichonyx oryzivorus*). Neurosci. Lett. 80, 229–234. (10.1016/0304-3940(87)90659-8) 3683981

[124] Semm P, Beason RC 1990 Responses to small magnetic variation by the trigeminal system of the bobolink. Brain Res. Bull. 25, 735–740. (10.1016/0361-9230(90)90051-Z) 2289162

[125] Beason RC, Semm P 1996 Does the avian ophthalmic nerve carry magnetic navigational information? J. Exp. Biol. 199, 1241–1244. 9319100 10.1242/jeb.199.5.1241

[126] Mora CV, Davison M, Wild JM, Walker MM 2004 Magnetoreception and its trigeminal mediation in the homing pigeon. Nature 432, 508–511. (10.1038/nature03077) 15565156

[127] Freire R, Dunston E, Fowler EM, McKenzie GL, Quinn CT, Michelsen J 2012 Conditioned response to a magnetic anomaly in the Pekin duck (*Anas platyrhynchos domestica*) involves the trigeminal nerve. J. Exp. Biol. 215, 2399–2404. (10.1242/jeb.068312) 22723478

[128] Chernetsov N, Kishkinev D, Mouritsen H, Heyers D 2008 A long-distance migrant compensates for longitudinal displacement. Curr. Biol. 18, 188–190. (10.1016/j.cub.2008.01.018) 18249113

[129] Kishkinev D, Chernetsov N, Pakhomov A, Heyers D, Mouritsen H 2015 Eurasian reed warblers compensate for virtual magnetic displacement. Curr. Biol. 25, R822–R823. (10.1016/j.cub.2015.08.012) 26439333

[130] Kishkinev D, Chernetsov N, Heyers D, Mouritsen H 2013 Migratory reed warblers need intact trigeminal nerves to correct for a 1000 km eastward displacement. PLoS ONE 8, e65847 (10.1371/journal.pone.0065847) 23840374 PMC3694148

[131] Pakhomov A, Anashina A, Heyers D, Kobylkov D, Mouritsen H, Chernetsov N 2018 Magnetic map navigation in a migratory songbird requires trigeminal input. Sci. Rep. 8, 11075 (10.1038/s41598-018-30477-8) 30097604 PMC6086908

[132] Gagliado A, Ioalè P, Savini M, Wild M 2008 Navigational abilities of homing pigeons deprived of olfactory and trigeminally mediated information when young. J. Exp. Biol. 211, 2046–2051. (10.1242/jeb.017608) 18552292

[133] Gagliado A, Ioalè P, Savini M, Wild M 2009 Navigational abilities of adult and experienced homing pigeons deprived of olfactory or trigeminally mediated magnetic information. J. Exp. Biol. 212, 3119–3124. (10.1242/jeb.031864) 19749104

[134] Walcott C 1978 Anomalies in the Earth's magnetic field increase the scatter of pigeons’ vanishing bearings. In Animal migration, navigation, and homing (eds Schmidt-Koenig K, Keeton WT), pp. 143–151. Berlin, Germany: Springer.

[135] Kiepenheuer J 1986 A further analysis of the orientation behavior of homing pigeons released within magnetic anomalies. In Biophysical effects of steady magnetic fields (eds Maret G, Boccara N, Kiepenheuer J). pp. 148–153. Berlin, Germany: Springer.

[136] Beason RC, Semm P 1991 Two different magnetic systems in avian orientation. In Acta XXX Congr. Int. Ornithol (eds Bell BD, Cossee RO, Flux JEC, Heather BD, Hitchmough RA, Robertson CJR, Williamsson MJ), pp. 1813–1819. Wellington, New Zealand: New Zealand Orthithol. Congr. Trust Board.

[137] Lefeldt N, Heyers D, Schneider NL, Engels S, Elbers D, Mouritsen H 2014 Magnetic field-driven induction of ZENK in the trigeminal system of pigeons (*Columba livia*). J. R. Soc. Interface 11, 20140777 (10.1098/rsif.2014.0777) 25232052 PMC4191110

[138] Wu LQ, Dickman JD 2011 Magnetoreception in the avian brain in part mediated by inner ear lagena. Curr. Biol. 21, 418–423. (10.1016/j.cub.2011.01.058) 21353559 PMC3062271

[139] Wu LQ, Dickman JD 2012 Neural correlates of a magnetic sense. Science 336, 1054–1057. (10.1126/science.1216567) 22539554

[140] Mouritsen H, Heyers D, Güntürkün O 2016 The neural basis of long-distance navigation in birds. Annu. Rev. Physiol. 78, 10.1–10.22. (10.1146/annurev-physiol-021115-105054) 26527184

[141] Bingman VP, Ioalé P, Casini G, Bagnoli P 1988 Hippocampal ablated homing pigeons show a persistent impairment in the time taken to return home. J. Comp. Physiol. A 163, 559–563. (10.1007/BF00604909)

[142] Quinn TP, Merrill RT, Brannon EL 1981 Magnetic field detection in sockeye salmon. J. Exp. Biol. 21, 137–142.(10.1002/jez.1402170114)

[143] Marhold S, Wiltschko W, Burda H 1997 A magnetic polarity compass for direction finding in a subterranean mammal. Naturwissenschaften 84, 421–423. (10.1007/s001140050422)

[144] Wang Y, Pan Y, Parsons S, Walker MM, Zhang S 2007 Bats respond to polarity of a magnetic field. Proc. R. Soc. B 274, 2901–2905. (10.1098/rspb.2007.0904) PMC2288691 17848365

[145] Phillips JB 1986 Magnetic compass orientation in the Eastern red-spotted newt (*Notophthalmus viridescens*). J. Comp. Physiol. A 158, 103–109. (10.1007/BF00614524) 3723427

[146] Light P, Salmon M, Lohmann KJ 1993 Geomagnetic orientation of loggerhead sea turtles: evidence for an inclination compass. J. Exp. Biol. 182, 1–10.

[147] Phillips JB, Borland SC 1992 Wavelength specific effects of light on magnetic compass orientation of the eastern red-spotted newt *Notophthalmus viridescens*. Ethol. Ecol. Evol. 4, 33–42. (10.1080/08927014.1992.9525348)

[148] Lohmann KJ, Lohmann CMF 1993 A light-independent magnetic compass in the leatherback sea turtle. Biol. Bull. 185, 149–151. (10.2307/1542138) 29300602

[149] Lohmann KJ, Lohmann CMF, Ehrhart LM, Bagley DA, Swing T 2004 Geomagnetic map used in sea-turtle navigation. Nature 428, 909–910. (10.1038/428909a) 15118716

